# Preliminary response to Janus kinase inhibition with baricitinib in chronic atypical neutrophilic dermatosis with lipodystrophy and elevated temperatures (CANDLE)

**DOI:** 10.1186/1546-0096-13-S1-O31

**Published:** 2015-09-28

**Authors:** G Montealegre, A Reinhardt, P Brogan, Y Berkun, A Zlotogorski, D Brown, P Chira, L Gao, J Dare, S Schalm, R Merino, D Chapelle, H Kim, S Judd, M O'Brien, A Almeida De Jesus, Y Kim, B Kost, Y Huang, S Paul, A Brofferio, C-C Lee, C Hadigan, T Heller, C Minniti, K Rother, R Goldbach-Mansky

**Affiliations:** 1 NIH/NIAMS, Bethesda, USA; 2 Children's Hospital of Omaha/UNMC, USA, USA; 3 Great Ormond Street Hospital, London, UK, UK; 4 Hadassah Medical Center, Jerusalem, Israel, Israel; 5 Children's Hospital Los Angeles, Los Angeles, CA, USA; 6 Riley Hospital for Children, Indianapolis, IN, USA; 7 University of Arkansas for Medical Sciences, Little Rock, AK, USA; 8 Arkansas Children's Hospital, Little Rock, AK, USA; 9 Dr. von Hauner Children's Hospital, Germany, USA; 10 University Hospital La Paz, Madrid, Spain, USA; 11 NIH/NIDDK, Bethesda, MD, USA; 12 NIH/CC/RDM, Bethesda, MD, USA; 13 NIH/NHLBI, Bethesda, MD, USA; 14 NIH/NIC, Bethesda, MD, USA; 15 NIH/NIAID, Bethesda, MD, USA

## Background

CANDLE is a novel autoinflammatory syndrome presenting early in infancy with attacks of fever, panniculitis, arthritis, myositis, lipodystrophy, cytopenias, dyslipidemia and growth retardation. CANDLE is caused by mutations in a number of proteasome-associated genes including *PSMA3*, *PSMB4*, *PSMB8* and *PSMB9*. Elevated serum IP-10 (CXCL10) levels and gene expression studies show a prominent “interferon (IFN) signature”. These findings and the successful *in vitro* blocking studies with Janus kinases (JAKs) inhibitors, raised the question whether IFN could be a therapeutic target in CANDLE.

## Objectives

The objective of this compassionate use study (NCT01724580) is to determine whether the treatment with baricitinib (JAK1/JAK2 inhibitor) results in the reduction of the mean Autoinflammatory Diary Score (ADS) to below 0.5 (ranges 0-4) and to determine whether treatment with baricitinib allows reduction of steroid doses by at least 50% in patients receiving steroids at baseline.

## Methods

Patients with CANDLE are eligible to participate in the study. Statistical analyses were performed using a paired t-test to compare baseline to last clinic visit data. Baricitinib dosing is based on a defined dose-escalation scheme. Patients who are unresponsive to treatment may have their dose escalated. Demographics, vital signs, safety laboratories, ADS, adverse events (AEs) and prednisone doses are captured at each visit.

## Results

To date, 12 CANDLE patients have been enrolled. One patient discontinued from the study due to lack of efficacy and development of debilitating avascular necrosis. All patients have been followed for at least 6 months (mean 1.7 years). The mean ADS decreased significantly (1.3 (SD± 0.8) at baseline, 0.3 (SD± 0.3) at the time of their last visit, p <0.005). The mean total prednisone dose of 14 mg/day (SD± 8.5) decreased by 73% to 3.8 mg/day (SD± 3.6). Four patients discontinued prednisone completely. Myositis has improved in 5 out of 6 patients and signs of bone marrow immunosuppression have improved in all but 1 patient (subject#1008), with increases of platelets, absolute lymphocyte counts (p <0.05) and hemoglobin. Seventeen serious adverse events (SAEs) were reported in 4 patients (presumed Pneumocystis jiroveci pneumonia, avascular necrosis, urinary tract, port cath-related, rotavirus, and C. difficile infections). The most common adverse events were upper respiratory infections, two patients have developed anemia. One patient has been treated successfully with IV iron.

The mean dose of baricitinib at last patient visit was 8.5 mg/day (SD± 2.1).

## Conclusion

Preliminary data in 11 CANDLE patients treated with baricitinib are encouraging and suggest that targeting IFN signaling with a JAK1/JAK2 inhibitor may be a successful therapeutic strategy for CANDLE patients, and possibly other IFN mediated autoinflammatory disorders.

